# Southern Medical Reports

**Published:** 1851-11

**Authors:** 


ART. V.—*Southern Medical Reports;* consisting of general and special reports on the medical topography, meteorology, and prevalent diseases of the following States: Louisiana, Alabama, Mississippi, North Carolina, South Carolina, Georgia, Florida, Arkansas, Tennessee, Texas, and California. Edited by E. D. Fenner, M. D., of New Orleans. Vol. ii. 1850.

We cordially thank Dr. Fenner for this renewed contribution to medical science. His labors are well directed, and should command the cordial support of his medical brethren throughout the Union.

In order to give our readers something of an idea of what Dr. Fenner is doing in the publication of this *annual* volume, we subjoin a copy of the table of contents:

*Reports from Louisiana.*

Report on the General Aspect of the Weather, the Condition of the Streets, the Stage of the River, and the Principal Diseases that prevailed in the City of New Orleans during the year 1850, in the form of a Monthly Journal. By the Editor.

Annual Report of the New Orleans and Lafayette Board of Health, for the year 1850.

Special Report on the Fevers of New Orleans, in the year 1850. By the Editor.

Special Report on Epidemic Cholera in the City of New Orleans during the year 1850. By the Editor.

Report upon the Meteorology, Vital Statistics and Hygiene of the State of Louisiana. By E. H. Barton, M. D.

On the Hydrography of the Mississippi River, for the year 1850. By Professor Caleb C. Forshey.

Report on the Medical Topography, Meteorology and Diseases of Trinity, La., and its vicinity, during the year 1850. By Andrew R. Kilpatrick, M. D.

On the Medical Topography and Diseases of the Parish of De Soto, La., with an Account of an Epidemic of Typhoid Fever which prevailed in Mansfield in the year 1850. By R. T. Gibbs, M. D.

On the Sanitary Condition of New Orleans, as illustrated by its Mortuary Statistics. By J. C. Simonds, M. D.

Special Report on Lead Poisoning in the City of New Orleans. By the Editor.

Report on the New Orleans Charity Hospital. By the Editor.

United States Marine Hospital—P. B. McKelvey, Surgeon.

State Medical Society of Louisiana. By the Editor.

Medical Department of the University of Louisiana—Session of 1850 and 1851.

*Reports from Alabama.*

Notes on the Topography, Sanitary Condition and Vital Statistics of Mobile, Ala. By Geoage A. Ketchum, M. D.

On the Diseases of Mobile, for the year 1850. By Wm. H. Anderson, M. D.

On the Climate and Diseases of Huntsville, Ala., and its vicinity, for the year 1850. By Jno. Y. Bassett, M. D.

Proceedings of the Medical Association of the State of Alabama.

On the Topography and Diseases of Lownsboro' and its vicinity, during the year 1850. By H. V. Wooten, M. D.

Remarks on the Good Effects of Large Doses of the Sulphate of Quinine in Continued Fever. By Thomas Fearn, M. D.

*Reports from Georgia.*

On the Vital Statistics of Hancock County, Georgia.—By E. M. Pendleton, M. D.

On the Dengue in the Cities of Savannah and Augusta. By Drs. R. D. Arnold and H. F. Campbell.

*Reports from South Carolina.*

An Account of the Epidemic Fever on Sullivan's Island in the Summer of 1850. By John B. Porter, M. D., Surgeon U. S. A.

A History of the Epidemic Dengue, as it prevailed in Charleston in the Summer of 1850. By Samnel Henry Dickson, M. D.

A History of the Break-Bone Fever—an Epidemic which prevailed in Charleston in the Summer of 1850. By Wm. T. Wragg, M. D.

On the Mortality of Charleston. By John L. Dawson, M. D.

*Reports from North Carolina.*

On the Topography, Prevailing Diseases, Epidemics and Public Institutions in the City of Raleigh, N. C., and the Surrounding Country. By W. H. McKee, M. D.

On the Vital Statistics of Middle North Carolina. By W. H. McKee, M. D.

*Reports from Mississippi.*

The Diseases and Physical Peculiarities of the Negro Race. By Samuel A. Cartwright, M. D.

On the Hygiene of Cotton Plantations, and the Management of Negro Slaves. By Thomas Affleck, Esq.

On the Dengue of Woodville, Miss. By A. C. Holt, M. D.

*Reports from Arkansas.*

On the Medical Topography and Diseases of Fort Gibson, Arkansas. By Richard H. Coolidge, Assistant Surgeon U. S. A.

*Reports from Texas.*

On the Climate, etc., of a Portion of Texas. By Ashbel Smith, Esq.

*Reports from California.*

On the Topography, Climate and Diseases of California. By Thomas M. Logan, M. D.

A History of the Epidemic Cholera that prevailed in Sacramento City in the Autumn of 1850.

Land Scurvy—its Pathology, Symptoms, Causes and Treatment; Poisonous Properties of Food and Water in a state of Fermentation or Putrefaction.

There are many of these reports of a highly practical character, and display a creditable degree of ability. The editor who has contrived to gather in his cause such an able body of assistants as Dr. Fenner has secured, deserves the approbation and encouragement of the medical profession. The work is published at two dollars and fifty cents per annum, a contribution to which, we think Dr. Fenner's enterprise is entitled from every medical man in the country. We sincerely hope that Dr. Fenner will be amply remunerated by his medical brethren for his well-directed labors—if *he* does not deserve well of those who feel interested in the advancement of medical science, we do not know who may present a claim.

